# Diversity, Taxonomy, and Pathogenicity of Members of *Fusarium tricinctum* Species Complex Associated with Wild Rosaceae Fruits

**DOI:** 10.3390/jof12050333

**Published:** 2026-05-02

**Authors:** Asanka Madhushan, Paul W. J. Taylor, Ahmed Mahmoud Ismail, Jian-Kui Liu, Sajeewa S. N. Maharachchikumbura

**Affiliations:** 1 Center for Informational Biology, School of Life Science and Technology, University of Electronic Science and Technology of China, Chengdu 611731, China; asankakwm@gmail.com; 2 Faculty of Science, The University of Melbourne, Parkville, VIC 3010, Australia; paulwjt@unimelb.edu.au; 3 Pests and Plant Diseases Unit, College of Agricultural and Food Sciences, King Faisal University, Al-Ahsa 31982, Saudi Arabia; amismail@kfu.edu.sa

**Keywords:** 4 new taxa, fruit rot, plant pathogens, phylogeny, *Sordariomycetes*

## Abstract

This study investigated *Fusarium* species associated with seven wild relatives of four economically important Rosaceae fruits in Sichuan Province, China, including wild strawberry (*Fragaria* sp. and *Potentilla indica*), wild raspberry (*Rubus rosaefolius*), wild cherry (*Prunus* sp., *Maddenia* sp. and *Prunus leveilleana*), and wild apple (*Malus kansuensis*). Based on multi-gene phylogenetic analyses and morphological characteristics, seven *Fusarium* species within the *Fusarium tricinctum* species complex (FTSC) were identified. Among these, four are described as new species (*F. fragariae*, *F. potentillae*, *F. pruni* and *F. fructicola*), while the remaining three represent new host records (*F. avenaceum*, *F. diversisporum* and *F. paeoniae*). In addition, phylogenetic and morphological evidence indicated that *F. rosiradicicola* is conspecific with *F. diversisporum*. Prioritizing the oldest epithet, we synonymized *F. rosiradicicola* under *F. diversisporum*. The pathogenicity of the isolates was evaluated on both their wild hosts and the corresponding cultivated fruits using detached, wound-inoculated assays. All tested isolates produced symptoms, showing pathogenic potential under experimental conditions. This study shows that selected wild Rosaceae fruits harbor several members of the FTSC and provides preliminary evidence of cross-host susceptibility under experimental conditions. However, further field-based investigations and non-wound inoculation studies are required to clarify their ecological roles, natural host susceptibility, and potential relevance in cultivated systems.

## 1. Introduction

Rosaceae is an economically important family comprising a variety of edible fruits and ornamentals, as well as timber, medicinal, and nutraceutical plants [1]. The family includes around 3000 diverse plant species, most of which are distributed in temperate regions [2]. In China, approximately 950 Rosaceae species are reported to occur, of which about 55% are endemic to the region [3]. Rosaceae fruits play a major role in the fruit industry of the country, as China is the world’s major producer of apples [4], pears [5], and strawberries [6]. Owing to their economic significance, several studies on pathogenic fungi affecting Rosaceae fruits have been conducted in China [7,8,9,10,11,12]. However, fungal pathogens associated with wild Rosaceae fruits remain poorly explored, despite their potential to act as reservoirs of pathogenic diversity and possible sources of emerging diseases in cultivated systems [13].

Sichuan Province, located in southwest China, is recognized as one of the hotspots of Rosaceae diversity [14]. Furthermore, studies on wild fruit resources in Sichuan Province have indicated that the Rosaceae is notably dominant among other families [15,16]. Among wild fruits of the Rosaceae, we selected wild strawberries (*Fragaria* spp.) and strawberry-like fruits (*Potentilla* spp.), apples (*Malus* spp.), cherries (*Prunus* spp.), and raspberries (*Rubus* spp.). In China and globally, there are relatively few studies on pathogens affecting these fruits. Niu et al. [17] investigated brown rot pathogens of stone and pome fruit trees in the wild forests of Xinjiang, China, and reported that *Monilinia laxa* and *Monilinia fructigena* are responsible for brown rot in *Malus sieversii*, while *Monilinia laxa* causes brown rot in *Prunus pseudocerasus*. Sir et al. [18] documented *Colletotrichum acutatum* causing anthracnose in *Potentilla indica* fruits. Ciui et al. [19] reported *Botrytis cinerea* as the causal agent of gray mold in *Rubus idaeus*, and Koponen et al. [20] recorded *Peronospora sparsa* (*Peronospora rubi*) as the causal agent of dry berry disease in wild *Rubus* species. However, reports of *Fusarium* species associated with these wild Rosaceae fruits are particularly scarce.

*Fusarium* is a well-known genus comprising globally important plant, animal, and human pathogens [21]. The genus comprises approximately 400 phylogenetically distinct species distributed across 20 species complexes and three undefined monophyletic lineages [22]. Among these, the *Fusarium tricinctum* species complex (FTSC) represents a phylogenetically distinct but relatively understudied group [23]. At present, 21 species are accepted within the FTSC [22]. Members of this complex have been reported as phytopathogens causing root rot in legumes [7,24,25] and other economically important crops [26,27,28], dry rot in potato [29], and head blight in wheat [28]. In addition, FTSC members have been associated with diseases of the domesticated species of the selected Rosaceae hosts. For example, *F. avenaceum* has been reported to cause apple core rot [30] and postharvest decay [31], as well as root rot in strawberry [32] and cane disease in raspberry [33]. *Fusarium acuminatum* has been associated with postharvest rot of Chinese cherry [34], while *F. tricinctum* has been reported to cause wilting disease in apple trees [35]. Moreover, Cheng et al. [36] documented xylem browning and dieback caused by *F. avenaceum* and *F. tricinctum* in wild apple forests in China. Despite these reports, studies focusing on fruit-associated infections by FTSC members remain limited.

In this study, we investigate the diversity and taxonomy of FTSC members associated with selected wild Rosaceae fruits in Sichuan Province using morphological and multi-locus phylogenetic analyses. In addition, we assess the pathogenic potential of selected isolates on both wild fruits and their corresponding cultivated fruits through controlled inoculation assays to partially fulfill Koch’s postulates. This study contributes to a better understanding of FTSC diversity in wild fruit systems, particularly from a taxonomic perspective, and provides preliminary insights into their potential interactions with cultivated Rosaceae hosts.

## 2. Materials and Methods

### 2.1. Sample Collection, Isolation, and Morphological Examination

Samples were collected from wild habitats in Sichuan Province, between May and October 2023. Symptomatic fruits of wild strawberries (*Fragaria* spp.) and strawberry-like fruits (*Potentilla* spp.), apples (*Malus* spp.), cherries (*Prunus* spp.), and raspberries (*Rubus* spp.) were detached from the plants and placed in paper envelopes until returned to the laboratory for further examination. The diseased fruits were surface-sterilized by immersing them in 75% ethanol for 2 min. After sterilization, the fruits were rinsed three times with sterile distilled water and dried using sterilized blotting papers. Tissue samples from the infected margins were cut into 3–4 mm^2^ pieces and incubated on potato dextrose agar (PDA; Oxoid Ltd., Wade Road, Basingstoke, Hants, RG24 8PW, UK) at 25 °C in the dark. After 2–3 days, the hyphal tips of the resulting fungi were transferred to fresh PDA and single-spore cultures were obtained following the method described by Leslie and Summerell [37]. Colony characteristics and growth rates were recorded from 7-day-old pure cultures incubated on fresh PDA and oatmeal agar (OA; Solarbio Science & Technology Co., Ltd., Beijing, China) at 25 °C. All the plates were supplemented with Tetracycline (60 mg/L) to control bacterial contaminations. Microscopic structures were observed using a Nikon ECLIPSE Ni-U microscope (Tokyo, Japan) and measured with Nikon NIS-elements documentation imaging 5.21.00 (Tokyo, Japan). Photographs were taken with a DS-Ri2 digital camera (Tokyo, Japan) and processed using Adobe Photoshop 22.0 (Adobe Inc., San Jose, CA, USA).

The ex-holotype specimens were deposited as dried cultures in the Herbarium of the University of Electronic Science and Technology (HUEST), Chengdu, China. The living ex-type cultures were deposited in the China General Microbiological Culture Collection Center (CGMCC), Beijing, China, with all additional living cultures and preservations stored in the University of Electronic Science and Technology Culture Collection (UESTCC), Chengdu, China. The taxonomic descriptions of the new taxa were registered in MycoBank.

### 2.2. DNA Extraction, Amplification, and Sequencing

Fresh fungal mycelia were obtained from 7-day-old cultures grown on PDA, and genomic DNA was extracted using the Trelief™ Plant Genomic DNA Kit (TSINGKE Biotech, Shanghai, China), following the instructions provided by the manufacturer. Five gene regions including ITS, *cal*, *rpb2*, *tef1*, and *tub2* were amplified by performing Polymerase Chain Reaction (PCR) using the primer pairs and conditions listed in Table 1.

The PCR mixture (30 µL) consisted 15 µL of 2× Flash PCR MasterMix (CoWin Biosciences, Taizhou, China), 11 µL of double-distilled water (ddH_2_O), 2 µL of DNA template, and 1 µL of each forward and reverse primer. PCR products were visualized using 1% agarose gel electrophoresis, and sequenced (Sangon Biotech, Shanghai, China). The newly generated sequences in this study are deposited in the NCBI GenBank.

### 2.3. Sequence Alignment and Phylogenetic Analysis

The chromatograms were checked for quality and assembled into consensus sequences using Seqman Pro 11.1.0 (DNASTAR, Inc., Madison, WI, USA). The isolates were preliminarily identified by comparing the ITS sequences against the NCBI database using BLASTn search (https://blast.ncbi.nlm.nih.gov/Blast.cgi (accessed on 27 November 2025)). Gene sets and reference sequences for the FTSC (Table 2) were downloaded from the NCBI nucleotide database. The sequence alignment was performed using MAFFT 7 online (https://mafft.cbrc.jp/alignment/server/ accessed on 27 November 2025). The alignment was manually checked and edited when needed using Aliview [47] and further trimmed using trimAl [48] with the “-gt 0.5” option. The alignments of each gene were combined using Phylosuite 1.2.3 software [49,50].

Maximum likelihood (ML) and Bayesian inference (BI) analyses were conducted to resolve the phylogeny. ML analysis was performed on the CIPRES Science Gateway platform [51] using RAxML-HPC2 on ACCESS with the GTR + GAMMA nucleotide evolution model and 1000 bootstrap replicates [52,53]. The best-fit evolution models for each gene were determined using the jModelTest2 on ACCESS in the CIPRES Gateway. BI analyses were conducted using MrBayes 3.2.6 [54] with one million generations, sampling a tree every 200 generations, and discarding 25% of the sampled trees as burn-in. The resulting phylogenetic trees were visualized with FigTree 1.4.0 and edited using Adobe Illustrator 2020 (Adobe Systems Inc., Lehi, UT, USA).

**Table 2:** GenBank accession numbers of *Fusarium* strains belonging to the *Fusarium tricinctum* species complex (FTSC). Newly generated sequences are indicated in bold, and ex-type strains are marked with “T” after the strain number. A dash (–) indicates that no sequence is available.

Species	Strain	GenBank Accession Numbers					Reference
		cal	ITS	tef1-α	tub2	rpb2	
*F. acuminatum*	LC13791	–	MW016644	MW620105	MW533990	MW474630	[55]
	LC13799	–	MW016652	MW620113	MW533998	MW474638	
*F*. *alpinum*	CGMCC 3.20818 ^T^	–	MW016689	MW620150	MW534035	MW474675	[55]
	LC6034	–	MW016686	MW620147	MW534032	MW474672	
	LC6037	–	MW016687	MW620148	MW534033	MW474673	
	LC2854	–	MW016685	MW620146	MW534031	MW474671	
*F. avenaceum*	CBS 408.86 ^T^	–	–	MW928836	–	MG282401	[22]
	GUCC 191095.1	OR043730	MZ724838	OR043880	OR043932	OR043825	[56]
	LC13801	–	MW016655	MW620116	MW534001	MW474641	[55]
	LC13802	–	MW016656	MW620117	MW534002	MW474642	
	LC13804	–	MW016658	MW620119	MW534004	MW474644	
	**UESTCC 25.0266**	**PX781469**	**PX776786**	**PX781496**	**PX781516**	**–**	**This study**
	**UESTCC 25.0267**	**PX781470**	**PX776787**	**PX781497**	**PX781517**	**PX781482**	
	**UESTCC 25.0268**	**PX781471**	**PX776788**	**PX781498**	**PX781518**	**PX781483**	
	**UESTCC 25.0269**	**PX781472**	**PX776789**	**PX781499**	**PX781519**	**PX781484**	
*F. californicum*	145796 ^T^	–	MK880138	MK878579	MK878574	MK878569	[57]
	BL24	–	MK880134	MK878575	MK878570	MK878565	
	BL28	–	MK880136	MK878577	MK878572	MK878567	
*F. campestre*	CBS 148994 ^T^	ON960621	ON951731	ON960701	ON960669	ON960685	[58]
	KG508	ON960633	ON951743	ON960713	ON960681	ON960697	
*F. chongqingense*	CGMCC 3.20821 ^T^	–	MW016677	MW620138	MW534023	MW474663	[55]
	LC13813	–	MW016675	MW620136	MW534021	MW474661	
	LC13814	–	MW016676	MW620137	MW534022	MW474662	
*F. citricola*	CPC27805 ^T^	–	LT746245	LT746197	–	LT746310	[59]
	CPC 27067	–	LT746242	LT746194	–	LT746307	
	CPC 27069	–	LT746243	LT746195	–	LT746308	
*F. dendranthematis*	ZHKUCC 24-0772 ^T^	–	–	PP983158	PP983185	PP983195	[60]
	ZHKUCC 24-0773	–	–	PP983159	PP983186	PP983196	
*F. diversisporum*	KNUF 21 F39	OP186043	–	OP186041	–	–	[61]
	BBA 11129	MZ921621	–	MZ921930	–	MZ921801	[22]
	**UESTCC 25.0270**	**PX781473**	**PX776790**	**PX781500**	**PX781520**	**PX781485**	**This study**
	**UESTCC 25.0271**	**PX781474**	**PX776791**	**PX781501**	**PX781521**	**PX781486**	
	**UESTCC 25.0272**	**PX781475**	**PX776792**	**PX781502**	**PX781522**	**–**	
	**UESTCC 25.0273**	**PX781476**	**PX776793**	**PX781503**	**PX781523**	**–**	
*F. diversisporum (*≡*F. rosiradicicola)*	CGMCC3.25482	OR043761	–	OR043914	OR043959	OR043858	[56]
	GUCC 191073.1	OR043763	–	OR043916	OR043961	OR043860	
	GUCC 191098.1	OR043766	–	OR043918	OR043964	OR043863	
*F. flavoides*	CGMCC 3.28711 ^T^	–	PV020684	PV050414	–	PV023180	[62]
*F. flocciferum*	CBS 821.68 ^T^	MZ921622	–	MW928837	–	MW928824	[63]
	CBS 147837	MZ921600	MZ890558	–	–	MZ921780	
	CBS 143231	MZ921598	MG386078	MG386159	MW534026	MG386149	[64]
	JW14005	–	MG386079	MG386160	–	MG386150	
***F. fragariae***	**UESTCC 25.0280 ^T^**	**PX781480**	**PX776800**	**PX781509**	**PX781529**	**–**	**This study**
	**UESTCC 25.0281**		**PX776801**	**PX781510**	**PX781530**	**–**	
*F. gamsii*	CBS 143610 ^T^	–	LT970824	LT970788	–	LT970760	[65]
	CBS 143609	–	LT970823	LT970787	–	LT970759	
*Fusarium* sp.	CGMCC 3.28976 ^T^	–	PV818710	PX659944	PX659950	PX659937	NCBI
	UESTCC 25.0264	–	PX410664	PX659949	PX659955	PX659943	
*F. iranicum*	CBS 143608 ^T^	–	LT970821	LT970785	–	LT970757	[65]
*F. meitneriae*	MST FP1765 ^T^	–	–	PP475466	–	PP475464	[66]
*F. paeoniae*	CGMCC 3.20817 ^T^	–	MW016681	MW620142	MW534027	MW474667	[55]
	LC13815	–	MW016679	MW620140	MW534025	MW474665	
	LC7358	–	MW016683	MW620144	MW534029	MW474669	
	**UESTCC 25.0274**	**PX781477**	**PX776794**	**PX781504**	**PX781524**	**–**	**This study**
	**UESTCC 25.0275**	**PX781478**	**PX776795**	**PX781505**	**PX781525**	**PX781487**	
	**UESTCC 25.0276**	**–**	**PX776796**	**PX781506**	**–**	**PX781488**	
	**UESTCC 25.0277**	**–**	**PX776797**	**PX781507**	**PX781526**	**PX781489**	
*F. paeoniae*	**UESTCC 25.0278**	**–**	**PX776798**	**PX781508**	**PX781527**	**–**	**This study**
	**UESTCC 25.0279**	**PX781479**	**PX776799**	**PX781504**	**PX781528**	**PX781490**	
*Fusarium* sp.	CGMCC 3.29115 ^T^	–	PX561138	PX508521	PX508519	PX508516	NCBI
***F. potentillae***	**UESTCC 25.0282 ^T^**	**–**	**PX776802**	**PX781511**	**PX781531**	**PX781491**	**This study**
	**UESTCC 25.0283**	**–**	**PX776803**	**PX781512**	**PX781532**	**PX781492**	
***F. pruni***	**UESTCC 25.0284 ^T^**	**–**	**PX776804**	**PX781513**	**PX781533**	**PX781493**	**This study**
	**UESTCC 25.0285**	**–**	**PX776805**	**PX781514**	**PX781534**	**PX781494**	
	**UESTCC 25.0286**	**–**	**PX776806**	**PX781511**	**PX781535**	**–**	
*F. reticulatum*	CBS 473.76 ^T^	–	–	MW928841	–	–	[22]
*F. rosendophyticum*	CGMCC3.25480 ^T^	–	MZ724841	–	OR043955	OR043855	[56]
	GUCC 190163.2	–	OR034269	–	OR043956	OR043856	
***F. fructicola***	**UESTCC25.0287 ^T^**	**PX781481**	**PX776807**	**PX781515**	**PX781536**	**PX781495**	**This study**
	**UESTCC 25.0288**	**–**	**PX776808**	**–**	**PX781537**	**–**	
*F. sinense*	CBS 122710 ^T^	–	EF531229	EF531235	EF531241	–	[67]
	CBS 122711	–	EF531230	EF531238	EF531242	–	
*F. torulosum*	NRRL 22748 ^T^	–	OL832305	OL772877	–	JX171615	[23]
	NRRL 52772	–	OL832315	OL772887	–	MH582377	
	JW 24001	–	MZ890423	MZ921918	MZ921822	MZ921788	[63]
*F. tricinctum*	CBS 253.50	–	–	KR071775	–	MW928823	[68]
	CBS 393.93 ^T^	–	HM068317	AB674263	–	JX171629	[69]
	LC13819	–	MW016693	MW620154	MW534039	MW474679	[55]
*F. concolor* (Outgroup)	NRRL 13994 ^T^	–	–	MH742650	–	MH742569	[70]

### 2.4. Pathogenicity Assays

For pathogenicity testing, one representative *Fusarium* strain per fruit was selected; however, two strains were included for *F. avenaceum*, *F. diversisporum*, and *F. paeoniae* due to their isolation from multiple fruits. The strains tested were *F. avenaceum* (UESTCC 25.0268 and UESTCC 25.0267), *F. diversisporum* (UESTCC 25.0270 and UESTCC 25.0271), *F. fragariae* (UESTCC 25.0280), *F. paeoniae* (UESTCC 25.0274 and UESTCC 25.0279), *F. potentillae* (UESTCC 25.0282), *F. pruni* (UESTCC 25.0284), and *F. fructicola* (UESTCC 25.0287). Healthy, mature, detached fruits from each wild host (except *Maddenia* sp., *Rubus rosaefolius*, and *Malus kansuensis*, due to unavailability) and cultivated fruits (strawberry, raspberry, cherry, and apple) were used for pathogenicity tests. Fruits were surface-sterilized with 75% ethanol for 2 min, rinsed twice with sterile distilled water, and dried using sterile blotting paper. Each fruit was wounded to a depth of approximately 1 mm using a sterile syringe needle to facilitate pathogen infection by overcoming plant defenses, and ensure reproducibility under controlled conditions [71,72]. Mycelial plugs (5 mm in diameter) taken from the margins of 7-day-old colonies were placed onto each wound, with the mycelium facing the fruit tissue. For negative controls, sterile PDA plugs were placed on similarly wounded fruits. Depending on fruit size, one to three inoculation sites were made per fruit, with three fruits used per treatment. All inoculated fruits were incubated in moist chambers at 25 °C for 7 days. The experiment was conducted twice. Fruits were examined daily for symptom development. Upon symptom appearance, fungi were re-isolated from symptomatic tissues and re-identified based on morphological characteristics and ITS sequence data to fulfill Koch’s postulates.

## 3. Results

### 3.1. Phylogenetic Analyses

**Figure 1:**
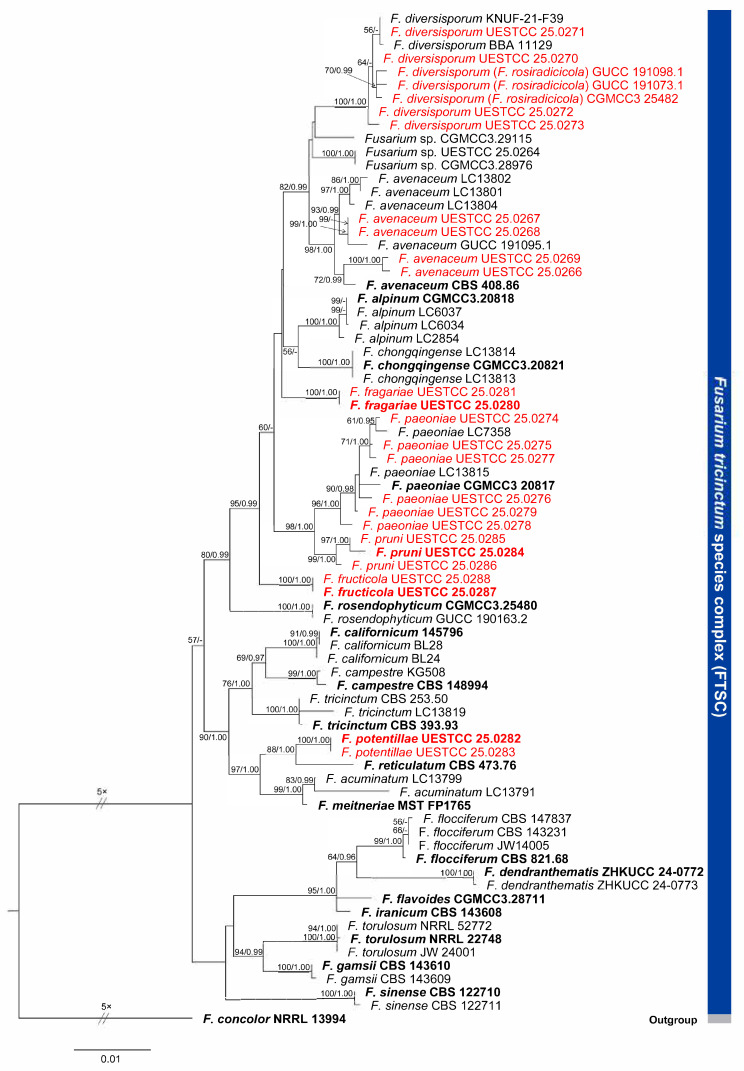
Phylogram of the *Fusarium tricinctum* species complex inferred from combined *cal*, ITS, *rpb2*, *tef1*, and *tub2* loci. The tree is rooted to *Fusarium concolor* NRRL 13994 (*F.* concolor species complex). Numbers at the nodes are RAxML bootstrap ≥50% and MrBayes posterior probability ≥ 0.90. Isolates of the ex-type are in bold. Isolates from the current study are indicated in red.

The phylogenetic analysis of the FTSC was performed by combining *cal*, ITS, *rpb2*, *tef1*, and *tub2* sequence data from 76 strains, including 23 isolates from the present study and *F. concolor* (NRRL 13994) as the outgroup (Figure 1).

The combined five-locus dataset (*cal*: 1–586; ITS: 587–1115; *rpb2*: 1116–2923; *tef1*: 2924–3583; and *tub2*: 3584–4122) comprised 877 distinct patterns and 37.16% undetermined characters or gaps. The best-fit evolution models for *cal*, ITS, *rpb2*, *tef1*, and *tub2* were K80, K80 + I, rNef + I + G, TrNef + G, and TrNef + G, respectively. The best-scoring ML tree (lnL = −12,393.780897), with support values from ML and Bayesian analyses at the node, is shown in Figure 1.

### 3.2. Taxonomy

Based on combined phylogenetic analyses and morphological comparisons, the 23 isolates represented seven species within the FTSC, including four new species (*F. fragariae*, *F. potentillae*, *F. pruni*, and *F. fructicola*) and three known species (*F. avenaceum*, *F. diversisporum*, and *F. paeoniae*). In addition, *F. rosiradicicola* is synonymized with *F. diversisporum* based on phylogenetic placement, high sequence similarity, and morphological congruence.

**Figure 2:**
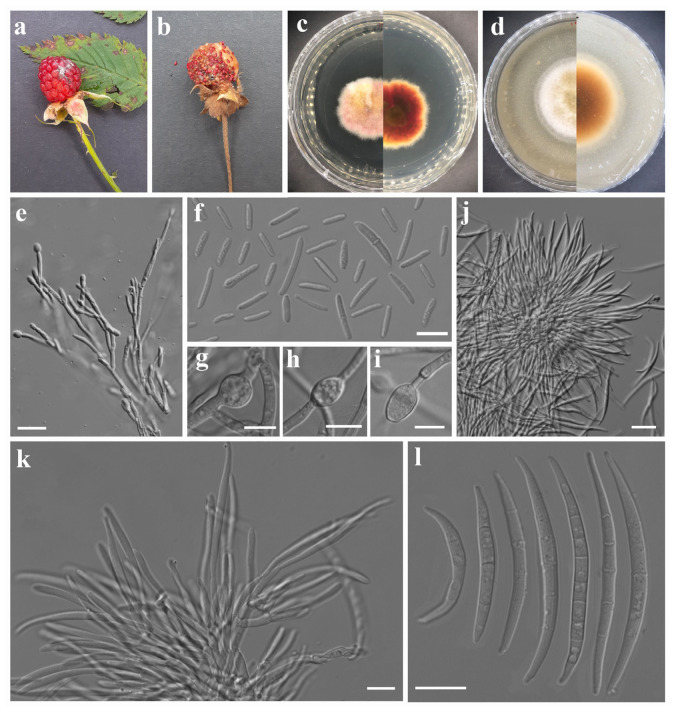
*Fusarium avenaceum*. (**a**) *Rubus rosaefolius* and (**b**) *Potentilla indica* fruits showing disease symptoms. Morphology of representative strain UESTCC 25.0266: (**c**) colony on PDA (surface and reverse). (**d**) Colony on OA (surface and reverse). (**e**) Aerial conidiophores and conidiogenous cells. (**f**) Microconidia. (**g**–**i**) Chlamydospores. (**j**,**k**) Sporodochial conidiophores and conidiogenous cells. (**l**) Macroconidia. Scale bars: (**f**–**i**,**k**,**l**) = 10 μm; (**e**,**j**) = 20 μm.

***Fusarium avenaceum*** (Fr.) Sacc., Syll. fung. (Abellini) 4: 713 (1886). Figure 2.

MycoBank: MB161610

Asexual morph: *Sporodochial conidiophores* branched, verticillate, dense. *Sporodochial conidiogenous cells* ampulliform to subcylindrical 12–15 × 2–3 µm (av. 13.56 × 2.63 µm, n = 15). *Sporodochial conidia* falcate and fusiform, elongate, and slightly curved with tapering apices; base poorly developed foot-shaped or well-developed foot-shaped; apex curved; hyaline, 2- to 7-septate, smooth- and thin-walled; 2-septate conidia: 21–24 × 3–4 µm (av. 22.22 × 3.47 µm, n = 10); 3-septate conidia: 22–30 × 3–5 µm (av. 25.7 × 4.13 µm, n = 10); 4-septate conidia: 32–46 × 3–5 µm (av. 39.06 × 3.82 µm, n = 10); 5-septate conidia: 39–60 × 3–5 µm (av. 47.53 × 3.99 µm, n = 10); 6-septate conidia: 44–54 × 3–4 µm (av. 50.40 × 3.77 µm, n = 8); 7-septate conidia: 58–63 × 3–4 µm (av. 60.55 × 3.67 µm, n = 7). *Aerial micro-conidiophores* polyphialides with multiple conidiogenous loci. *Aerial micro-conidiogenous cells* monophialidic, ampulliform to cylindrical, 15–24 × 2–4 µm (av. 19.08 × 3.10 µm, n = 12), smooth- and thin-walled. *Microconidia* abundant, straight, allantoid or slightly curved fusiform, hyaline, non-septate or 3-septate, smooth- and thin-walled; non-septate conidia: 6–13 × 2–4 µm (av. 10.01 × 2.97 µm, n = 20); 1-septate conidia: 13–21 × 3–4 µm (av. 16.88 × 3.37 µm, n = 15); 2-septate conidia: 17–21 × 4–5 µm (av. 18.74 × 4.15 µm, n = 5); 3-septate conidia: 19–20 × 3–4.5 µm (av. 19.28 × 4.08 µm, n = 5). *Chlamydospores* globose to subglobose, hyaline, smooth-walled, single, terminal or intercalary, 7–14 µm (av. 10.21 µm, n = 5). Sexual morph: Not observed.

*Culture characteristics*: Colonies on PDA attaining 30 mm diameter after 5 days in the dark at 25 °C, surface pink, yellow near the center, raised, floccose, white irregular margin, and reverse reddish orange with yellowish white margin. On OA attaining 45 mm diameter after 5 days, white, dull yellow near the center, velvety, raised with entire margin, aerial mycelium moderate, and reverse yellowish brown with white margin.

*Material examined*: China. Sichuan Province: Deyang City, Swan Forest Farm, 31°16′19″ N, 103°56′59″ E, elevation 1351.66 m, 26 May 2023, on *Rubus rosaefolius* fruits showing fruit rot symptoms, A. Madhushan RB111 (Dry culture HUEST 25.0236), Living culture UESTCC 25.0268; *ibid*., RB112 (Dry culture HUEST 25.0237), Living culture UESTCC 25.0269; *ibid*., on *Potentilla indica* fruits showing fruit rot symptoms, A. Madhushan ST108 (Dry culture HUEST 25.0234), Living culture UESTCC 25.0266; *ibid*., ST121 (Dry culture HUEST 25.0235), Living culture UESTCC 25.0267.

*Notes*: In the phylogenetic analysis, our isolates grouped with *F. avenaceum* strains including the neotype (CBS 408.86). In addition, our strains share morphological characteristics similar to those of *F. avenaceum* [29,52]. Therefore, based on multi-locus phylogeny and morphology, we introduce our isolates as *F. avenaceum*. This species has been reported as both a saprobe and a pathogen from a wide range of substrates, including soils, economically important cereals, ornamental plants, vegetables, and diverse fruit crops. Documented hosts include wheat, barley, carnations, *Eustoma grandiflorum*, *Hydrangea macrophylla*, broccoli, Douglas fir, lentils, linseed, apple, strawberry, raspberry, cherry, peach, and nectarine [30,31,32,37,60]. To our knowledge, this is the first record of *F. avenaceum* on *Rubus rosaefolius* and *Potentilla indica*.

**Figure 3:**
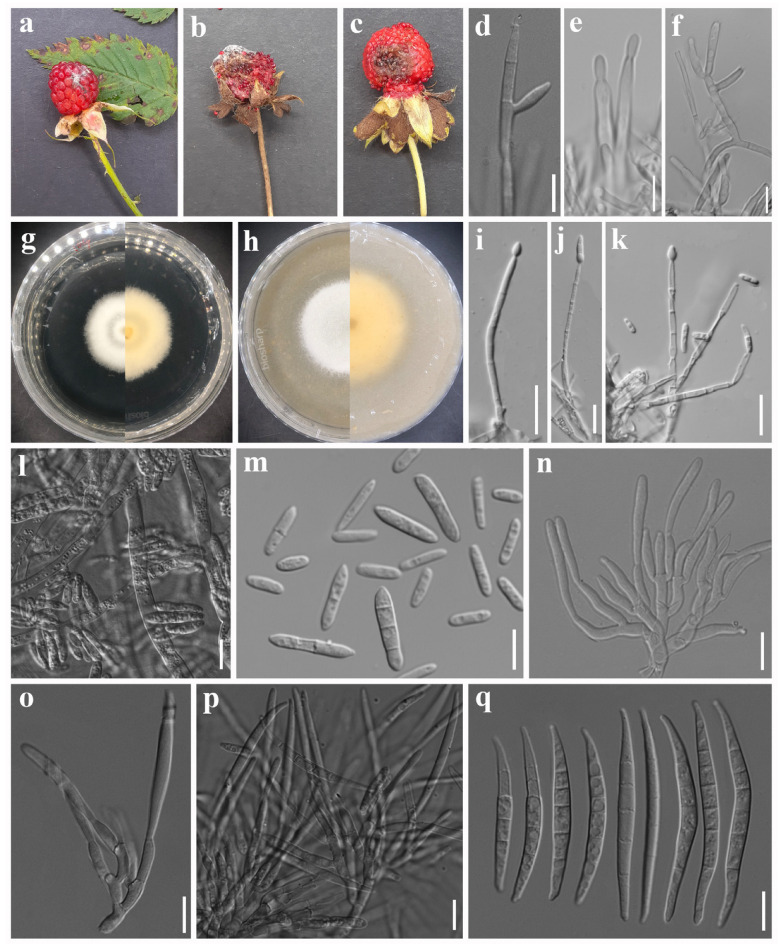
*Fusarium diversisporum.* (**a**) *Rubus rosaefolius* [image reused from Figure 2a due to co-infection] and (**b**,**c**) *Potentilla indica* fruits showing disease symptoms. Morphology of representative strain UESTCC 25.0273: (**d**–**f**) aerial macro-conidiophores and conidiogenous cells. (**g**) Colony on PDA (surface and reverse). (**h**) Colony on OA (surface and reverse). (**i**–**l**) Aerial micro-conidiophores and conidiogenous cells. (**m**) Microconidia. (**n**–**p**) Sporodochial conidiophores and conidiogenous cells. (**q**) Macroconidia. Scale bars: 10 μm.

***Fusarium diversisporum*** Sherb., Mem. Cornell Univ. Agric. Exp. Stn 6: 161 (1915). Figure 3.

≡***Fusarium rosiradicicola*** H. Zhang & Y.L. Jiang, in Zhang, Zeng, Wei, Jiang & Zeng, Mycosphere 14(1): 2125 (2023).

MycoBank: MB194315

Asexual morph: *Sporodochial conidiophores* unbranched or branched, bearing terminal and lateral verticils of monophialides. *Sporodochial conidiogenous cells* ampulliform to subcylindrical, 10–17 × 3–4 µm (av. 12.53 × 3.34 µm, n = 15). *Sporodochial conidia* fusiform, elongate, and unequally curved with tapering apices; base papillate, nonfoot-shaped to well-developed, foot-shaped; apex curved, or long and tapered; hyaline, 3-septate to 6-septate, smooth- and thin-walled; 3-septate conidia: 30–35 × 4–5 µm (av. 32.14 × 4.15 µm, n = 5); 4-septate conidia: 40–50 × 3–5 µm (av. 44.03 × 3.60 µm, n = 10); 5-septate conidia: 49–55 × 3–4 µm (av. 50.82 × 3.78 µm, n = 10); 6-septate conidia: 52–57 × 3–4 µm (av. 54.55 × 3.54 µm, n = 5). *Aerial conidiophores* erect or prostrate on substrate mycelium, branched or unbranched, or reduced to monophialides, forming laterally or terminally on aerial mycelium. *Aerial conidiogenous cells* monophialidic, doliiform to ampulliform, 11–19 × 2–3 µm (av. 15.41 × 2.89 µm, n = 15), smooth- and thin-walled. *Aerial macroconidia* conidia indistinguishable from sporodochial conidia. *Microconidia* abundant, allantoid, fusiform, or oval, straight, hyaline, non-septate to 3-septate, smooth- and thin-walled; non-septate conidia: 8–18 × 3–4 µm (av. 11.30 × 2.93 µm, n = 20); 1-septate conidia: 12–23 × 3–4 µm (av. 17.01 × 3.54 µm, n = 15); 2-septate conidia: 21–23 × 4–5 µm (av. 21.99 × 4.09 µm, n = 5); 3-septate conidia: 20–21 × 4–5 µm (av. 20.43 × 4.10 µm, n = 3). Sexual morph: Not observed.

*Culture characteristics*: Colonies on PDA attaining 37 mm diameter after 5 days in the dark at 25 °C, surface white, raised ring near the center, cottony, entire margin, and reverse yellow. On OA attaining 55 mm diameter after 5 days, white, no ring formation, velvety, moderately raised, entire margin, and reverse yellow.

*Material examined*: CHINA. GUIZHOU PROVINCE: Liupanshui City, 25°52′52″ N, 104°33′59″ E, elevation 20,247 m, 4 August 2020, from healthy roots and stems of *Rosa roxburghii* (Rosaceae), H. Zhang (HGUP 190168), ex-type Living culture: GUCC 190168.1 = CGMCC3.25482; other Living cultures: GUCC 190100.1, GUCC 191129.1, GUCC 190145.1, GUCC 191098.1, and GUCC 190194.1; *ibid*., Guiyang City, 27°4′50″ N, 106°29′50″ E, elevation 1184 m, 22 April 2020, healthy stems of *Rosa roxburghii* (Rosaceae), *H. Zhang*, Living cultures: GUCC 191009.1 and GUCC 191073.1 [56]; *ibid*., SICHUAN PROVINCE: Deyang City, Swan Forest Farm, 31°16′19″ N, 103°56′59″ E, elevation 1351.66 m, 26 May 2023, on *Potentilla indica* fruits showing fruit rot symptoms, A. Madhushan ST102 (Dry culture HUEST 25.0238), Living culture UESTCC 25.0270; *ibid*., ST101 (Dry culture HUEST 25.0240), Living culture UESTCC 25.0272; *ibid*., ST116 (Dry culture HUEST 25.0241), Living culture UESTCC 25.0273; *ibid*., on *Rubus rosaefolius* fruits showing fruit rot symptoms, A. Madhushan RB104 (Dry culture HUEST 25.0239), Living culture UESTCC 25.0271.

*Notes*: *Fusarium diversisporum* was first described by Sherbakoff (1915) from *Solanum tuberosum*. However, the holotype specimen (CBP-007430) lacks sequence data. Subsequently, isolate CBS 795.70, obtained from *Prunus domestica*, was designated as the authentic reference strain for *F. diversisporum* [22]. *Fusarium rosiradicicola* was described by Zhang et al. [56] from roots and stems of *Rosa roxburghii*. However, their phylogenetic analysis did not include *F. diversisporum*. Later phylogenetic analyses of the FTSC showed that strains of *F. rosiradicicola*, including the type strain CGMCC 3.25482, are monophyletic with strains of *F. diversisporum* ([60]; this study). Furthermore, NCBI BLAST comparisons revealed high sequence similarity between *F. rosiradicicola* (CGMCC 3.25482) and *F. diversisporum* CBS 795.70, with *cal*, *rpb1*, *rpb2*, and *tef1* sequence identities of 99.5% (593/596; no gaps), 99.89% (1741/1743; gaps 1/1743), 99.89% (913/914; gaps: 1/914), and 99.07% (636/642; gaps: 4/642), respectively. In addition, *F. rosiradicicola* and *F. diversisporum* display similar macro- and micro-morphological characteristics [56,73]. Based on phylogenetic position, high sequence similarity, and morphological similarities, we treat *F. rosiradicicola* as a synonym of *F. diversisporum*.

In the present phylogenetic analysis, the isolates from this study were grouped within the clade containing *F. diversisporum* (CBS 795.70 = BBA 11129). Morphologically, our strains share similarities with *F. diversisporum*, except for the presence of up to 3-septate microconidia and 6-septate macroconidia, which have not been reported in either CBS 795.70 [73] or CGMCC 3.25482 [56]. Therefore, based on both phylogenetic and morphological analyses, we identify our isolates as *F. diversisporum*. In addition to *Solanum tuberosum*, *Prunus domestica*, and *Rosa roxburghii*, *F. diversisporum* has also been reported from *Malus domestica* [61] and citrus (reported as *F. rosiradicicola*) [74]. To our knowledge, this is the first record of *F. diversisporum* infecting *Rubus rosaefolius* and *Potentilla indica*.

**Figure 4:**
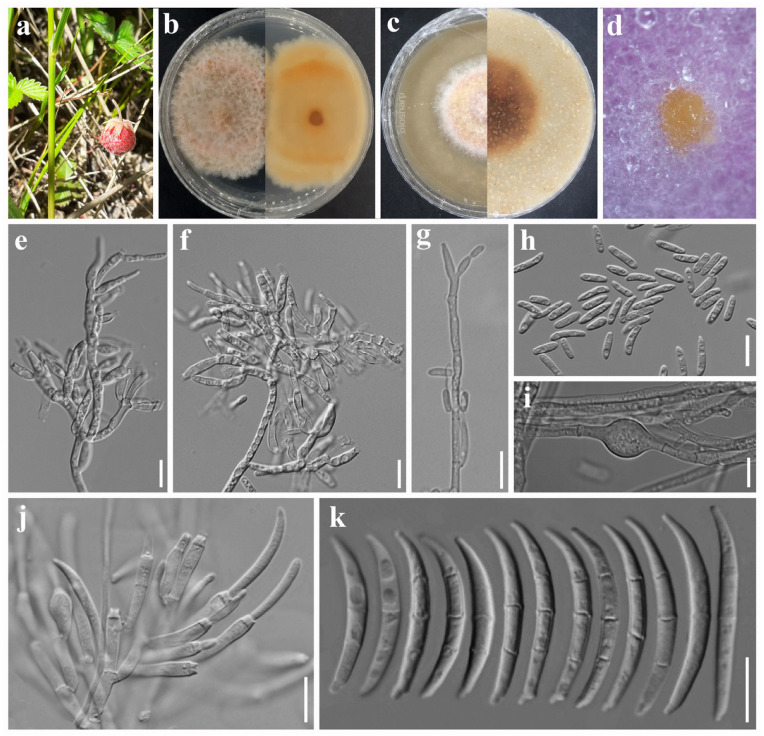
*Fusarium fragariae* (ex-type, UESTCC 25.0280). (**a**) Wild *Fragaria* sp., the host from which the fungus was isolated. (**b**) Colony on PDA (surface and reverse). (**c**) Colony on OA (surface and reverse). (**d**) Sporodochia on PDA. (**e**,**f**) Aerial macro-conidiophores and conidiogenous cells. (**g**) Aerial micro-conidiophores and conidiogenous cells. (**h**) Microconidia. (**i**) Chlamydospore. (**j**) Sporodochial conidiophores and conidiogenous cells. (**k**) Macroconidia. Scale bars: 10 μm.

***Fusarium fragariae*** Madhushan & Maharachch., sp. nov. Figure 4.

MycoBank: MB861720

*Typification*: China. Sichuan Province: Aba Prefecture, Xiaojin County, Majia Gou, 31°32′9″ N, 102°25′23″ E, elevation 3214.45 m, 5 July 2023, on *Fragaria* sp. fruits showing fruit rot symptoms, A. Madhushan ST201-1 (**holotype** represents ex-holotype HUEST 25.0248 metabolic inactive culture on PDA). Ex-type Living culture UESTCC 25.0280.

*Etymology*: The name refers to the host genus (*Fragaria*) from which the type was isolated.

Asexual morph: *Sporodochia* yellowish orange, abundant on PDA. *Sporodochial conidiophores* branched, bearing terminal and lateral verticils of monophialides. *Sporodochial conidiogenous cells* cylindrical to subcylindrical or doliiform, 11–18 × 2–3 µm (av. 14.45 × 2.63 µm, n = 20). *Sporodochial conidia* fusoid, dorsiventrally curved, tapering toward apices, or slender with no significant curvature; base well-developed foot-shaped; apex curved, or blunt; hyaline, 1–3-septate, smooth- and thin-walled; 1-septate conidia: 22–30 × 2–3 µm (av. 25.52 × 2.78 µm, n = 10); 2-septate conidia: 25–32 × 2–3 µm (av. 27.35 × 2.77 µm, n = 10); 3-septate conidia: 27–33 × 2–3 µm (av. 29.70 × 2.88 µm, n = 15). *Aerial conidiophores* erect or prostrate on substrate mycelium, branched or unbranched. *Aerial conidiogenous cells* monophialidic or polyphialides, doliiform, ampulliform, or cylindrical to subcylindrical, 7–13 × 2–4 µm (av. 9.31 × 2.96 µm, n = 15), smooth- and thin-walled. *Aerial macroconidia* indistinguishable from sporodochial conidia. Microconidia abundant, broadly ellipsoidal, allantoid to fusiform, hyaline, non-septate or 1-septate, smooth- and thin-walled; non-septate conidia: 7–12 × 3–4 µm (av. 9.43 × 2.57 µm, n = 25); 1-septate conidia: 13–16 × 2–3 µm (av. 14.5 × 2.65 µm, n = 8). *Chlamydospores* globose to subglobose, hyaline, thin-walled, intercalary, singly, 9–15 µm (av. 11.73 µm, n = 5) diameter. Sexual morph: Not observed.

*Culture characteristics*: Colonies on PDA attaining 28 mm diameter after 5 days in the dark at 25 °C, surface raised, floccose, creamy white center, surrounded by a pinkish ring and an outer creamy white irregular margin, and reverse yellowish orange center, surrounded by a reddish orange ring and an outer yellowish orange margin. On OA attaining 21 mm diameter after 5 days, surface pinkish, yellow ring surrounding the center, outer white margin, cottony, raised, entire margin, and reverse yellowish brown.

*Other specimens examined*: China. Sichuan Province: Aba Prefecture, Xiaojin County, Majia Gou, 31°32′9″ N, 102°25′23″ E, elevation 3214.45 m, 5 July 2023, on *Fragaria* sp. fruits showing fruit rot symptoms, A. Madhushan ST201-2 (Dry culture HUEST 25.0249), Living culture UESTCC 25.0281.

*Notes*: In the phylogenetic analysis, our strains formed a single lineage close to *Fusarium paeoniae*, *F. pruni*, *F. alpinum*, and *F. chongqingense* (Figure 1). Morphologically, our strains differ from *F. paeoniae* in having fewer septa in macroconidia (3–5 in *F. paeoniae* vs. 1–3 in *F. fragariae*) and microconidia (0–1(–3) in *F. paeoniae* vs. 0–1 in *F. fragariae*), and in producing chlamydospores, which are absent in *F. paeoniae* [55]. Our strains differ from *F. pruni* by microconidial size (5–25 × 2–4 µm in *F. pruni* vs. 7–16 × 2–4 µm in *F. fragariae*), and by having distinctly curved macroconidia with well-developed foot-shaped basal cells (this study). Compared to *F. alpinum*, our strains differ in macroconidial size (37.8 × 3.7 µm in *F. alpinum* vs. 29.70 × 2.88 µm in *F. fragariae*) and in possessing well-developed foot-shaped basal cells and chlamydospores, the latter of which are absent in *F. alpinum* [55]. Our strains differ from *F. chongqingense* by macroconidial size (25.7 × 4 µm in *F. chongqingense* vs. 29.70 × 2.88 µm in *F. fragariae*) and shape (slightly curved macroconidia with blunt basal cells in *F. chongqingense* vs. distinctly curved macroconidia with well-developed foot-shaped basal cells in *F. fragariae*), and by the presence of microconidia, which are not observed in *F. chongqingense* [55]. In addition, the nucleotide of *F. fragariae* (ST201) differs from *F. paeoniae* (CGMCC 3.20817) by 2.92% (565/582; gaps: 9/582) variations in *tef1*, and 1.6% (492/500; no gaps) variations in *tub2*; differs from *F. pruni* (CH207) by 1.55% (573/582; gaps: 1/582) variations in *tef1*, and 1.31% (533/540; gaps: 0/540) variations in *tub2*; differs from *F. alpinum* (CGMCC 3.20818) by 1.89% (571/582; gaps: 4/582) variations in *tef1*, and 1.37% (493/501; gaps: 1/501) variations in *tub2*; and differs from *F. chongqingense* (CGMCC 3.20821) by 2.23% (569/582; gaps: 2/582) variations in *tef1*, and 1.2% (495/501; gaps: 1/501) variations in *tub2*. Based on morphology, phylogeny, and sequence data, we introduce *F. fragariae* as a new species in the FTSC.

**Figure 5:**
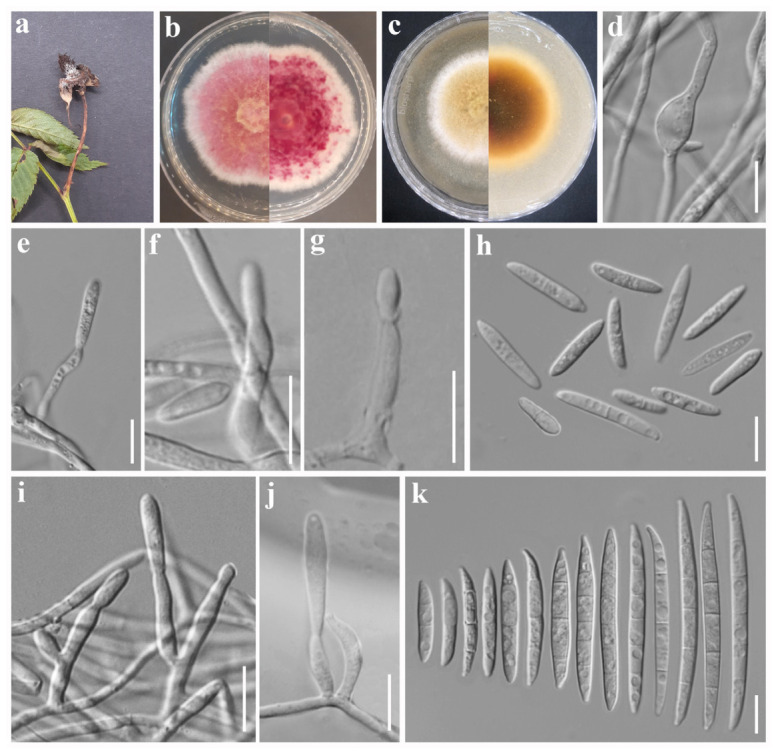
*Fusarium fructicola* (ex-type, UESTCC 25.0287). (**a**) *Rubus rosaefolius* fruit receptacle, sepals and peduncle showing disease signs and symptoms. (**b**) Colony on PDA (surface and reverse). (**c**) Colony on OA (surface and reverse). (**d**) Chlamydospore. (**e**–**g**) Aerial micro-conidiophores and conidiogenous cells. (**h**) Microconidia. (**i**,**j**) Aerial macro-conidiophores and conidiogenous cells. (**k**) Macroconidia. Scale bars: (**d**–**k**) = 10 μm.

***Fusarium fructicola*** Madhushan & Maharachch., sp. nov. Figure 5.

MycoBank: MB861726

*Typification*: China. Sichuan Province: Deyang City, Swan Forest Farm, 31°16′19″ N, 103°56′59″ E, elevation 1351.66 m, 26 May 2023, on *Rubus rosaefolius* fruit receptacle, A. Madhushan RB105-1 (**holotype** represents ex-holotype HUEST 25.0255 metabolic inactive culture on PDA), ex-type Living culture UESTCC 25.0287.

*Etymology*: Referring to the substrate (fruit) from which the type species was isolated.

Asexual morph: *Aerial conidiophores* erect or prostrate on substrate mycelium, branched or unbranched, or reduced to monophialides, forming laterally or terminally on aerial mycelium. *Aerial macro-conidiogenous cells* monophialidic, ampulliform to subcylindrical, 6–15 × 2–3 µm (av. 11.03 × 2.74 µm, n = 10). *Aerial micro-conidiogenous cells* monophialidic, ampulliform to subcylindrical, 13–14 × 2–3 µm (av. 13.43 × 2.67 µm, n = 5). *Macroconidia* fusiform, elongate, and not or slightly curved with tapering apices; base papillate nonfoot-shaped to well-developed foot-shaped; apex curved, or long and tapered; hyaline, 2-septate to 5-septate, smooth- and thin-walled; 2-septate conidia: 22–23 × 3–4 µm (av. 22.28 × 3.66 µm, n = 5); 3-septate conidia: 29–36 × 3–5 µm (av. 33.77 × 4.25 µm, n = 5); 4-septate conidia: 34–51 × 3–5 µm (av. 45.03 × 3.94 µm, n = 10); 5-septate conidia: 48–59 × 4–5 µm (av. 52.90 × 4.19 µm, n = 8). *Microconidia* abundant, fusiform to allantoid, elongate, straight, hyaline, non-septate to 1-septate, smooth- and thin-walled; non-septate conidia: 13–17 × 2–3 µm (av. 14.48 × 2.88 µm, n = 10); 1-septate conidia: 12–25 × 3–4 µm (av. 18.24 × 3.28 µm, n = 15). *Chlamydospores* subglobose, hyaline, thin-walled, intercalary, single, 7–10 µm (av. 8.84 µm, n = 15) diameter. Sexual morph: Not observed.

*Culture characteristics*: Colonies on PDA attaining 30 mm diameter after 5 days in the dark at 25 °C, surface purplish pink, yellowish raised mycelium at the central region, floccose, white irregular margin, and reverse purplish pink with a white color margin. On OA attaining 52 mm diameter after 5 days, surface dull yellow with a white margin, floccose, raised, entire margin, and reverse dark brown with yellowish white margin.

*Other specimens examined*: China. Sichuan Province: Deyang city, Swan Forest Farm, 31°16′19″ N, 103°56′59″ E, elevation 1351.66 m, 26 May 2023, on *Rubus rosaefolius* fruit receptacle, *A. Madhushan RB105-2* (Dry culture HUEST 25.0256), Living culture UESTCC 25.0288.

*Notes*: In the phylogenetic analysis, the two isolates obtained in this study formed a single lineage with 95% ML bootstrap support and 0.99 BYPP values, close to those of *F. rosendophyticum*, *F. paeoniae*, and *F. pruni* (Figure 1). Morphologically, our strains differ from *F. rosendophyticum* in macroconidial size (34.9 × 2.8 µm in *F. rosendophyticum* vs. 52.90 × 4.19 µm in *F. fructicola*), colony coloration (beige on PDA and gray on OA in *F. rosendophyticum* vs. purplish pink on PDA and dull yellow on OA in *F. fructicola*), and in the presence of septa in microconidia, which are absent in *F. rosendophyticum* [56]. Our strains differ from *F. paeoniae* by macroconidial length (45.2 µm in *F. paeoniae* vs. 52.90 µm in *F. fructicola*) and by the presence of chlamydospores, which are not observed in *F. paeoniae* [55]. Compared with *F. pruni*, our strains produce macroconidia with up to 5 septa, whereas *F. pruni* forms macroconidia with up to 3 septa (this study). In addition, the nucleotide of *F. fructicola* (UESTCC 25.0287) differs from *F. rosendophyticum* (CGMCC3.25480) by 2.12% (878/897; gaps: 5/897) variations in *rpb2*, and 3.04% (510/526; gaps: 0/526) variations in *tub2*; differs from *F. paeoniae* (CGMCC3.20817) by 2.60% (862/885; gaps: 9/885) variations in *rpb2*, 2.76% (598/615; gaps: 9/615) variations in *tef-1*, and 1.60% (492/500; no gaps) variations in *tub2*; and differs from *F. pruni* (CGMCC of 207) by 0.98% (912/921; gaps: 9/921) variations in *rpb2*, 1.30% (606/614; gaps: 1/614) variations in *tef-1*, and 1.85% (530/540; gaps: 0/540) variations in *tub2*. Based on morphology, phylogeny, and sequence data, we introduce *F. fructicola* as a new species in the FTSC.

**Figure 6:**
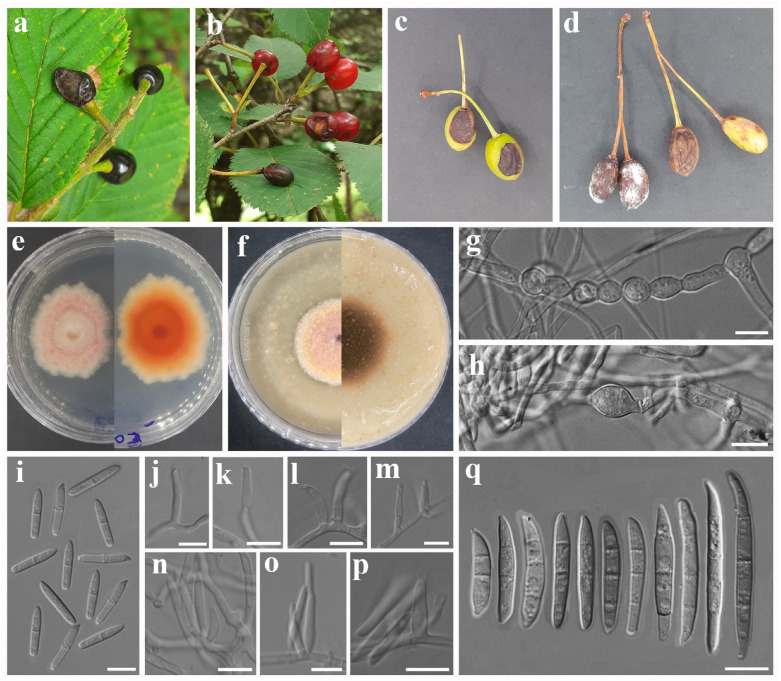
*Fusarium paeoniae.* (**a**) *Maddenia* sp., (**b**) *Prunus leveilleana*, (**c**) *Prunus* sp. and (**d**) *Malus kansuensis* fruits showing disease symptoms. Morphology of representative strain UESTCC 25.0279: (**e**) colony on PDA (surface and reverse). (**f**) Colony on OA (surface and reverse). (**g**,**h**) Chlamydospores. (**i**) Microconidia. (**j**–**p**) Aerial conidiophores and conidiogenous cells. (**q**) Macroconidia. Scale bars: 10 μm.

***Fusarium paeoniae*** M.M. Wang & L. Cai, in Wang, Crous, Sandoval-Denis, Han, Liu, Liang, Duan & Cai, Persoonia 48: 41 (2022). Figure 6.

MycoBank: MB842161

Asexual morph: *Aerial conidiophores* erect or prostrate on substrate mycelium, unbranched, or reduced to monophialides, forming laterally or terminally on aerial mycelium. *Aerial conidiogenous cells* monophialidic, doliiform, ampulliform to subcylindrical, 9–20 × 3–4 µm (av. 14.21 × 3.05 µm, n = 12), smooth- and thin-walled. *Aerial macroconidia* slender with no significant curvature, or fusiform, elongate, straight or slightly curved with tapering apices; base obtuse nonfoot-shaped, papillate nonfoot-shaped, or poorly developed foot-shaped; apex blunt or curved, 1–3-septate, hyaline, smooth- and thin-walled; 1-septate conidia: 22–23 × 3–4 µm (av. 21.61 × 3.14 µm, n = 5); 2-septate conidia: 20–26 × 3–4 µm (av. 22.71 × 3.54 µm, n = 10); 3-septate conidia: 24–42 × 3–5 µm (av. 30.21 × 3.84 µm, n = 10). *Microconidia* abundant, hyaline, elongate, cylindrical, rounded at base and pointed at apex, non-septate or 1-septate, smooth- and thin-walled; non-septate conidia: 8–17 × 3–4 µm (av. 11.90 × 3.25 µm, n = 10); 1-septate conidia: 13–20 × 3–4 µm (av. 16.92 × 3.22 µm, n = 10). *Chlamydospores* globose to subglobose, hyaline, thick-walled, intercalary, singly or multiple, 6–11 µm (av. 7.86 µm, n = 5) diameter. Sexual morph: Not observed.

*Culture characteristics*: Colonies on PDA attaining 22 mm diameter after 5 days in the dark at 25 °C, surface floccose, raised, central region yellowish-white, surrounded by a pinkish zone, margin white, irregular, and reverse reddish orange with a yellowish white margin. On OA attaining 41 mm diameter after 5 days, cottony, central region pinkish, surrounded by a thin yellowish zone, margin white, entire, and reverse dark brown.

*Material examined*: China. Sichuan Province: Aba Prefecture, Xiaojin County, Siguniang Mountain, 31°0′30″ N, 103°50′55″ E, elevation 3374.05 m, 4 July 2023, on *Prunus* sp. fruits showing fruit rot symptoms, A. Madhushan CH213 (Dry culture HUEST 25.0246), Living culture UESTCC 25.0278; *ibid*., Majia Gou, 31°32′9″ N, 102°25′23″ E, elevation 3214.45 m, 5 July 2023, on *Malus kansuensis* fruits showing fruit rot symptoms, A. Madhushan AP220 (Dry culture HUEST 25.0242), Living culture UESTCC 25.0274; *ibid*., Li County, Miyaluo town, 31°43′7″ N, 102°48′19″ E, elevation 2944.46 m, 6 July 2023, on *Maddenia* sp. fruits showing fruit rot symptoms, A. Madhushan CH222 (Dry culture HUEST 25.0244), Living culture UESTCC 25.0276; *ibid*., Li County, Miyaluo town, 31°43′7″ N, 102°48′19″ E, elevation 2944.46 m, 6 July 2023, on *Maddenia* sp. fruits showing fruit rot symptoms, A. Madhushan CH223 (Dry culture HUEST 25.0245), Living culture UESTCC 25.0277; *ibid*., LI County, Miyaluo town, 31°43′7″ N, 102°48′19″ E, elevation 2944.46 m, 6 July 2023, on *Prunus leveilleana* fruits showing fruit rot symptoms, A. Madhushan CH228 (Dry culture HUEST 25.0243), Living culture UESTCC 25.0275; *ibid*., Li County, Miyaluo town, 31°43′7″ N, 102°48′19″ E, elevation 2944.46 m, 6 July 2023, on *Prunus leveilleana* fruits showing fruit rot symptoms, A. Madhushan CH230 (Dry culture HUEST 25.0247), Living culture UESTCC 25.0279.

*Notes*: In the phylogenetic analysis, our isolates grouped with *F. paeoniae* strains, including the ex-type (CGMCC 3.20816). Morphologically, our strains are similar to *F. paeoniae* [55]. However, CGMCC 3.20816 produces 3–5-septate macroconidia and 0–3-septate microconidia, whereas our strains produce 1–3-septate macroconidia and 3–5-septate microconidia. Based on phylogenetic and morphological analyses, we designate our isolates as *F. paeoniae*. *Fusarium paeoniae* was first described by Wang et al. [55] from *Paeonia lactiflora*. In addition, *F. paeoniae* has also been reported from *Viscum album* subsp. austriacum [75] and *Fagus sylvatica* [76]. To our knowledge, this is the first record of *F. diversisporum* on *Maddenia* sp., *Prunus leveilleana*, *Prunus* sp. and *Malus kansuensis*.

**Figure 7:**
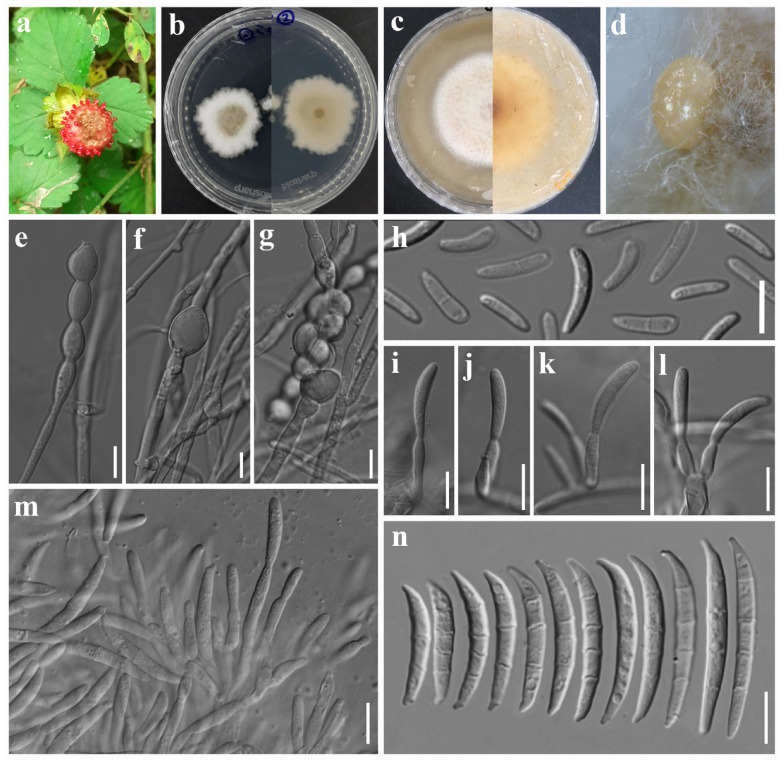
*Fusarium potentillae* (ex-type, UESTCC 25.0282). (**a**) *Potentilla indica* showing disease symptoms. (**b**) Colony on PDA (surface and reverse). (**c**) Colony on OA (surface and reverse). (**d**) Sporodochia on PDA. (**e**–**g**) Chlamydospores. (**h**) Microconidia. (**i**–**l**) Aerial conidiophores and conidiogenous cells. (**m**) Sporodochial conidiophores and conidiogenous cells. (**n**) Macroconidia. Scale bars: 10 μm.

***Fusarium potentillae*** Madhushan & Maharachch., sp. nov. Figure 7.

MycoBank: MB861721

*Typification*: China. Sichuan Province: Aba Prefecture, Li County, Liangtaigou town, 31°22′44″ N, 102°52′5″ E, elevation 2686.82 m, 7 July 2023, on *Potentilla indica* fruits showing fruit rot symptoms, A. Madhushan ST252-1 (**holotype** represents ex-holotype HUEST 25.0250 metabolic inactive culture on PDA), ex-type Living culture UESTCC 25.0282.

*Etymology*: The name refers to the host genus (*Potentilla*) from which the type was isolated.

Asexual morph: *Sporodochial conidiophores* branched, bearing terminal and lateral verticils of monophialides. *Sporodochial conidiogenous cells* monophialidic, subcylindrical to ampulliform, 7–20 × 3–4 µm (av. 11.05 × 3.18 µm, n = 25). *Sporodochial conidia* falcate, curved with parallel walls or unequally curved; base well-developed, foot-shaped; base well-developed, foot-shaped; apex hooked, or long and tapered; hyaline, 1-septate to 4-septate, smooth- and thin-walled; 1-septate conidia: 15–22 × 3–5 µm (av. 17.93 × 3.27 µm, n = 8); 2-septate conidia: 22–29 × 3–4 µm (av. 24.01 × 3.61 µm, n = 10); 3-septate conidia: 23–32 × 3–4 µm (av. 27.84 × 3.65 µm, n = 15); 4-septate conidia: 28–39 × 3–4 µm (av. 32.19 × 3.80 µm, n = 10). *Aerial conidiophores* erect or prostrate on substrate mycelium, reduced to monophialides forming laterally or terminally on aerial mycelium. *Aerial conidiogenous cells* subcylindrical to ampulliform, 7–16 × 2–3 µm (av. 9.87 × 3.06 µm, n = 15), smooth- and thin-walled. *Aerial macroconidia* indistinguishable from sporodochial conidia. *Microconidia* abundant, allantoid, slightly curved, hyaline, non-septate to 1-septate, smooth- and thin-walled; non-septate conidia: 7–17 × 2–3 µm (av. 11.78 × 2.97 µm, n = 10); 1-septate conidia: 13–18 × 3–4 µm (av. 15.18 × 3.14 µm, n = 10). *Chlamydospores* globose, subglobose to oval, hyaline, thin-walled, intercalary, single or multiple, 7–21 µm (av. 11.63 µm, n = 20) diameter. Sexual morph: Not observed.

*Culture characteristics*: Colonies on PDA attaining 21 mm diameter after 5 days in the dark at 25 °C, surface white, brownish yellow at the center, floccose, irregular margin, and reverse yellowish white. On OA attaining 51 mm diameter after 5 days, surface yellowish white, velvety, entire margin, and reverse yellowish brown.

*Other specimens examined*: China. Sichuan Province: Aba Prefecture, Li County, Liangtaigou town, 31°22′44″ N, 102°52′5″ E, elevation 2686.82 m, 7 July 2023, on *Potentilla indica* fruits showing fruit rot symptoms, A. Madhushan ST252-2 (Dry culture HUEST 25.0251), Living culture UESTCC 25.0283.

*Notes*: In the phylogenetic analysis, the two isolates from our study formed a sister clade with *Fusarium reticulatum*, with 90% ML bootstrap support and 1.00 BYPP values (Figure 1). An NCBI BLAST search of the *tef1* sequence against *F. reticulatum* CBS 473.76 showed a 1.44% bp (617/626; no gaps) difference from our isolate. Morphologically, our strains share many characteristics with *F. reticulatum* [22]; however, they differ by producing only 1–4-septate macroconidia, whereas *F. reticulatum* produces 0–5-septate macroconidia, and microconidia are absent in *F. reticulatum*. Based on these distinctions, we introduce our isolates as a new species within the FTSC.

**Figure 8:**
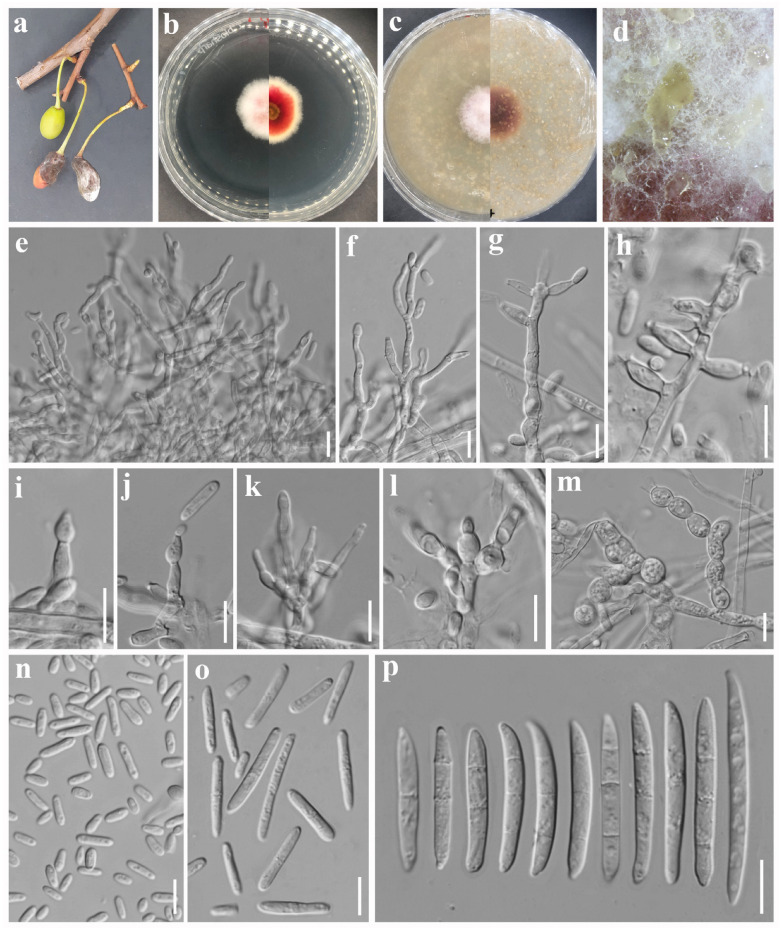
*Fusarium pruni* (ex-type, UESTCC 25.0284). (**a**) Wild *Prunus* sp. fruit showing disease symptoms. (**b**) Colony on PDA (surface and reverse). (**c**) Colony on OA (surface and reverse). (**d**) Sporodochia on PDA. (**e**–**l**) Sporodochial (micro-) conidiophores and conidiogenous cells. (**m**) Chlamydospores. (**n**,**o**) Microconidia. (**p**) Macroconidia. Scale bars: (**e**–**p**) = 10 μm.

***Fusarium pruni*** Madhushan & Maharachch., sp. nov. Figure 8.

MycoBank: MB861724

*Typification*: China. Sichuan Province: Aba Prefecture, Xiaojin County, Siguniang Mountain, 31°0′30″ N, 103°50′55″ E, elevation 3374.05 m, 4 July 2023, on *Prunus* sp. fruits showing fruit rot symptoms, A. Madhushan CH207 (**holotype** represents ex-holotype HUEST 25.0252 metabolic inactive culture on PDA), ex-type Living culture UESTCC 25.0284.

*Etymology*: The name refers to the host genus (*Prunus*) from which the type was isolated.

Asexual morph: *Sporodochia* yellowish, less abundant on PDA, and composed with microconidia. *Sporodochial conidiophores* irregularly branched, bearing lateral and terminal monophialides. *Sporodochial conidiogenous cells* monophialidic or polyphialidic, doliiform to ampulliform, 6–17 × 3–4 µm (av. 10.52 × 3.49 µm, n = 15). *Macroconidia* straight to moderately dorsiventrally curved, tapering toward the apex; base papillate nonfoot-shaped, or well-developed foot-shaped; apex curved, or long and tapered, sometimes irregularly swollen at bottom; hyaline, 1-septate to 3-septate, smooth- and thin-walled; 1-septate conidia: 27–28 × 3–4 µm (av. 27.30 × 3.60 µm, n = 8); 2-septate conidia: 27–35 × 3–4 µm (av. 29.65 × 3.73 µm, n = 10); 3-septate conidia: 29–36 × 3–4 µm (av. 32.11 × 3.9 µm, n = 15). *Microconidia* abundant, allantoid or oval, straight, some are elongated, hyaline, non-septate to 1-septate, smooth- and thin-walled; non-septate conidia: 5–14 × 2–3 µm (av. 10.41 × 2.90 µm, n = 20); 1-septate conidia: 12–25 × 3–4 µm (av. 17.31 × 3.37 µm, n = 15). *Chlamydospores* globose to subglobose, hyaline, thin-walled, intercalary, multiple, 6–10 µm (av. 7.94 µm, n = 15) diameter. Sexual morph: Not observed.

*Culture characteristics*: Colonies on PDA attaining 27 mm diameter after 5 days in the dark at 25 °C, surface purplish pink with a white margin, raised mycelial ring near the center, raised, floccose, irregular margin, and reverse reddish orange with white margin. On OA attaining 24 mm diameter after 5 days, surface pinkish with white mycelia on the top, floccose, raised, nearly entire margin, and reverse yellowish brown.

*Other specimens examined*: China. Sichuan Province: Aba Prefecture, Xiaojin County, Siguniang Mountain, 31°0′30″ N, 103°50′55″ E, elevation 3374.05 m, 4 July 2023, on *Prunus* sp. fruits showing fruit rot symptoms, A. Madhushan CH209 (Dry culture HUEST 25.0253), Living culture UESTCC 25.0285; *ibid*., CH211 (Dry culture HUEST 25.0254), Living culture UESTCC 25.0286.

*Notes*: In the phylogenetic analysis, three isolates from our study formed a lineage with 96% ML bootstrap support and 1.00 BYPP values, close to those of *F. paeoniae* (Figure 1). Our strains are morphologically distinct from other *Fusarium* spp. due to the abundance of microconidia in sporodochia (sporodochia are composed mainly of macroconidia in most *Fusarium* spp.). Our strains also differ from the closely related *F. paeoniae* in microconidial shape (oval to allantoid and 0–1-septate in *F. pruni* vs. ellipsoid to falcate and 0–3-septate in *F. paeoniae*) and in the presence of chlamydospores, which are not observed in *F. paeoniae* [55]. Based on these variations, we introduce our isolates as new species in the FTSC.

### 3.3. Pathogenicity

**Figure 9:**
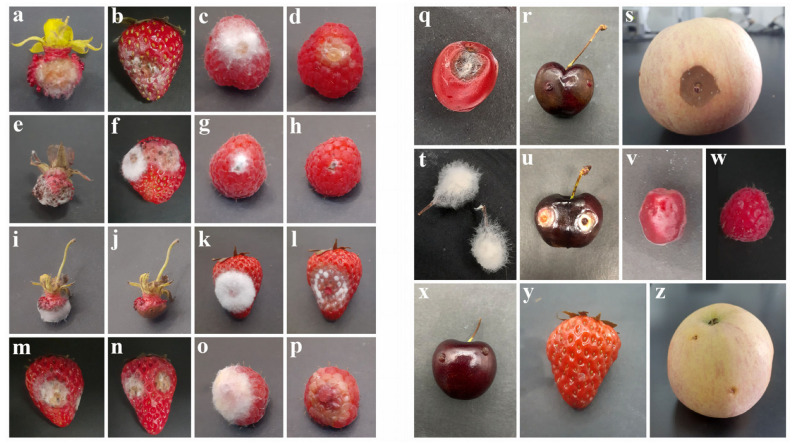
Symptoms on detached wild and cultivated fruits 3–5 days post-inoculation with *Fusarium* species under experimental conditions: *F. avenaceum* on *Potentilla indica* (**a**), strawberry (**b**), and raspberry (**c**,**d**); *F. diversisporum* on *Potentilla indica* (**e**), strawberry (**f**), and raspberry (**g**,**h**); *F. potentillae* on *Potentilla indica* (**i**,**j**) and strawberry (**k**,**l**); *F. fragariae* on strawberry (**m**,**n**); *F. fructicola* on raspberry (**o**,**p**); *F. paeoniae* on *Prunus levelina* (**q**), cherry (**r**), and apple (**s**); *F. pruni* on *Prunus* sp. (**t**) and cherry (**u**); control fruits: *Prunus levelina* (**v**), raspberry (**w**), cherry (**x**), strawberry (**y**), and apple (**z**).

All *Fusarium* species tested produced disease symptoms on both wild and cultivated fruits under the experimental conditions (Figure 9). The pathogenic potential of selected isolates was confirmed by observing symptoms in inoculated fruits and comparing them with the control treatment, in which no symptoms were observed. Symptoms started to appear 2–3 days after inoculation. Except for apple, all *Fusarium* species showed abundant mycelial growth on the fruit surface, and brown to yellow water-soaked lesions were observed after removal of the mycelial layer. *Fusarium paeoniae* produced brown, water-soaked lesions on apple fruits. Koch’s postulates were fulfilled, as the fungal isolates re-isolated from symptomatic tissues exhibited morphological characteristics and ITS sequences identical to the original isolates.

## 4. Discussion

We conducted a survey on fungi associated with symptomatic wild fruits in Sichuan Province, China. During this survey, 23 *Fusarium* strains were isolated from symptomatic fruits of seven wild Rosaceae hosts. Based on morphological characterization and phylogenetic analyses, these isolates were identified as seven *Fusarium* species. Among them, four are newly described (*F. fragariae*, *F. potentillae*, *F. pruni*, and *F. fructicola*), while the remaining three were identified as *F. avenaceum*, *F. diversisporum*, and *F. paeoniae*. Notably, all seven species belong to the FTSC, suggesting that members of this complex may be associated with Rosaceae hosts. Evidence from previous studies further supports this association, as some of the members of FTSC, including *F. californicum* [57], *F. rosendophyticum*, and *F. rosiradicicola* (synonymized here as *F. diversisporum*) [56], were originally described from Rosaceae hosts. In addition, *F. acuminatum* [77,78], *F. avenaceum* [79,80], *F. diversisporum* [22], and *F. tricinctum* [35,81] have been reported from various Rosaceae hosts. However, further studies are needed to determine whether these fungi share common genomic traits or have independently adapted to these hosts. Consistent with our findings, Talhinhas and Baroncelli [82] reported that Rosaceae harbor the highest number of host–species association records in *Colletotrichum*, further supporting the notion that certain fungal lineages may exhibit preferential associations with Rosaceae hosts.

Wild strawberries, also referred to as mock strawberries, comprise species belonging to the genera *Fragaria*, *Duchesnea*, and *Potentilla* [83,84,85]. During the survey, symptomatic *Fragaria* sp. and *Potentilla indica* were collected as wild strawberries. Fungal isolates obtained from these hosts included *F. avenaceum*, *F. diversisporum*, *F. fragariae* sp. nov. and *F. potentillae* sp. nov. Previous studies have reported the pathogenicity of *F. avenaceum* on cultivated strawberries, including root rot [32] and seedling infections [86]. Pastrana et al. [87] also isolated *F. avenaceum* from strawberry roots and crowns but found it to be non-pathogenic under their experimental conditions. However, *F. avenaceum* has not previously been reported in association with strawberry fruit rot. In the present study, detached, wound-inoculated fruit assays demonstrated that *F. avenaceum* and the newly described species induced disease symptoms on strawberry fruits under experimental conditions.

Symptomatic wild raspberry (*Rubus rosaefolius*) fruits yielded three FTSC members, including *F. avenaceum*, *F. diversisporum*, and *F. fructicola* sp. nov. Among them, *F. avenaceum* is a known causal agent of fruit rot in raspberries (*Rubus idaeus*) [79]. In addition, *F. avenaceum* has been reported to cause bud death, lateral wilt [88], and root diseases [89] in cultivated raspberries. According to our preliminary pathogenicity assays, *F. diversisporum* and *F. fructicola* were also shown to be capable of inducing disease symptoms on raspberry fruits under controlled conditions. Symptomatic fruits of different wild cherry species, including *Prunus* sp., *Maddenia* sp. (*Prunus* sp.), and *Prunus leveilleana*, yielded *F. paeoniae* and *F. pruni* sp. nov. as associated fungal species with fruit rot. While *F. pruni* is newly described in this study, *F. paeoniae* has not previously been reported in association with cherry fruits. Moreover, *F. paeoniae* was isolated from wild apple (*Malus kansuensis*) and has not been recorded in association with cultivated apples. Our preliminary pathogenicity assays indicated that these species can induce disease symptoms on cultivated cherry and apple fruits under experimental conditions.

These results provide preliminary evidence of pathogenic potential under artificial inoculation conditions and expand the known host associations of FTSC members. However, the use of wounded, detached fruits may overestimate host susceptibility, as this approach bypasses natural infection barriers and does not reflect field conditions [71]. These findings therefore suggest only possible cross-host pathogenic potential under experimental conditions rather than evidence of natural host shifts or disease emergence. Pathogenicity assessments under field or semi-field conditions are therefore required to better evaluate infection potential, and the role of interactions with other microorganisms remains to be further investigated. As large-scale fruit farming increasingly encroaches into natural habitats, and the consumption of wild fruits becomes more widespread, the proximity between wild and cultivated plants is likely to increase. Such conditions may increase opportunities for contact between fungal isolates associated with diseased wild hosts and cultivated crops. In this context, documenting fungal diversity and host associations in wild fruit systems is important for early detection and monitoring of potential pathogens.

This study provides a taxonomic framework for understanding FTSC diversity associated with wild Rosaceae fruits and offers preliminary insights into their pathogenic potential under experimental conditions. Fungal isolates associated with diseases of wild hosts may possess distinct pathogenicity-related traits, as wild plants often exhibit strong disease resistance [90], which can facilitate pathogen adaptation. Future research should prioritize integrative omics approaches, particularly comparative genomics and transcriptomics, to elucidate the genetic basis of pathogenic potential in these fungi, with emphasis on effector repertoires and avirulence (*Avr*) genes. Knowledge of *Avr* gene diversity and distribution can be integrated into plant breeding programs to identify, deploy, and pyramid corresponding resistance (R) genes [91,92], thereby improving the durability of disease resistance.

## Figures and Tables

**Table 1:** Primer pairs and PCR conditions used in the study.

Locus	Primers	PCR Amplification Protocol	References
ITS	ITS5/ITS4	94 °C for 90 s; 35 cycles of 94 °C for 45 s, 55 °C for 45 s, 72 °C for 1 min; 72 °C for 10 min; 10 °C on hold	[38]
*cal*	cal-228F/cal-2Rd, CL1/CL2A	94 °C for 90 s; 35 cycles of 94 °C for 45 s, 55 °C for 45 s, 72 °C for 1 min; 72 °C for 10 min; 10 °C on hold	[39,40,41]
*rpb2*	5f2/7cr	94 °C for 90 s; 35 cycles of 94 °C for 45 s, 52 °C for 45 s, 72 °C for 1 min; 72 °C for 10 min; 10 °C on hold	[42,43]
*tef-1*	EF1/EF2	94 °C for 90 s; 35 cycles of 94 °C for 45 s, 55 °C for 45 s, 72 °C for 1 min; 72 °C for 10 min; 10 °C on hold	[39,44,45]
*tub2*	T1/T2	94 °C for 90 s; 35 cycles of 94 °C for 45 s, 55 °C for 45 s, 72 °C for 1 min; 72 °C for 10 min; 10 °C on hold	[46]

## Data Availability

All sequence data are available in NCBI GenBank following the accession numbers mentioned in the manuscript.
